# An Experimental Study of Temperature Effect on Properties of Nitride Layers on X37CrMoV51 Tool Steel Used in Extrusion Aluminium Industry

**DOI:** 10.3390/ma13102311

**Published:** 2020-05-17

**Authors:** Rafał Hubicki, Maria Richert, Marcel Wiewióra

**Affiliations:** 1 Grupa Kęty S.A. S.A., str. Kościuszki 111, 32-650 Kęty, Poland; rhubicki@grupakety.com; 2 AGH University of Science and Technology, str. Gramatyka 10, 30-067 Kraków, Poland; marcelwiewiora@gmail.com

**Keywords:** nitriding, extrusion dies, hardness, structure

## Abstract

The paper concerns the effect of annealing time and temperature on the properties of the nitride layer on X37CrMoV51 tool steel used in the extrusion aluminium industry. Samples made from X37CrMoV51 steel were hardened and tempered, and then nitrided at 530 °C. After nitriding, the samples were annealed in a furnace at 470 °C for 8 h, 12 h, 24 h, 30 h and 60 h, and additionally for 20 h at 270 °C. The samples were tested for structure, hardness and abrasion immediately after nitriding and again after annealing. It was found that annealing the nitrided samples leads to degradation of the nitride layer, accounting for the decrease of hardness. The annealing of the samples at 470 °C, over 12 h causes a decrease in mean hardness value from 1176 HV to 1103 HV, and annealing the samples over 30 h at this temperature leads to a decrease in hardness to 964 HV. The changes in nitrogen content in the white (compound) and diffusion layers and the resulting consequences of changes in phase composition and properties were evaluated. Annealing over 30 h at 470 °C caused the white layer to disappear and the average nitrogen content in the diffusion layer to decrease to the level of about 5–6 at%.

## 1. Introduction

Nitriding of tool surfaces is one of the most commonly used types of heat treatment of tool steels. The nitriding process increases the hardness of the surface layers as well as resistance to abrasion and corrosion [1,2,3,4].

The properties achieved by nitriding may change due to the use of the dies and their annealing. During the extrusion process the die is exposed to high temperatures that affect the surface condition. Before the dies are placed in the press, they are heated, which also affects the condition of the nitride layer.

Dies for extrusion of aluminium alloys are subjected to repeated regeneration by secondary nitriding, which results in multiple high temperature treatments. The need for regeneration results from the abrasion of the nitrided layer from the surface of the calibrating bearing of the dies during their exploitation. 

Nitriding consists in diffusing nitrogen into the surface layer of steel. It is carried out in the temperature range 500–650 °C in an ammonia atmosphere. Above 400 °C, the ammonia decomposes according to the NH_3_ → 3H + N reaction. Atomic nitrogen diffuses into the steel and forms nitride phases. At the temperature of 591 °C, the nitride layer consists of three phases: ε-Fe_2_N nitride, γ′-Fe_4_N nitride and nitrogen ferrite, which contains 0.01% nitrogen at room temperature. At the temperature of 600–650 °C, the γ phase is formed, which, when slowly cooled down to the temperature of 591 °C, transforms into eutectoid ε + γ′ [5,6,7,8,9].

The hardness of the nitride layer reaches the level of 1100–1200 HV and should maintain this level when reheated to working temperatures of 500–600 °C. The nitride layer of tempered steel is wear-resistant.

The nitriding process, which lasts from about 20 to 50 h, produces a layer with a thickness of 0.2–0.4 mm. Increasing the nitriding temperature causes an increase in the thickness of the nitride layer, however, it reduces its hardness, which adversely affects the resistance to abrasion. Literature data indicate that lower nitriding temperature favours high hardness of the nitride layer and good resistance against erosion and corrosion resistance [1,2,3]. 

The effect of nitrogen presence in the atomic structure of steel is the formation of nitrides and the interstitial location of nitrogen in the crystal lattice. Depending on the concentration of nitrogen, different stoichiometric nitride compositions can be found. Nitrides as compounds, such as titanium nitride [10] or boron nitride [11], are characterized by high hardness and refractory properties, and are used in the production of cutting tool blades, refractory laboratory vessels and protective coatings. Gallium nitride was used in the production of blue laser [12]. Like some oxides, nitrides can absorb hydrogen and are considered for hydrogen storage, e.g., lithium nitride [13].

Depending on the concentration of nitrogen, there are different stoichiometric nitride compositions in steels. Apart from nitrides formed as a result of the connection of nitrogen and iron, such as Fe_2_N, and Fe_4_N, CrN nitrides are also formed in steels containing chromium [14,15,16,17,18,19]. The formation of a CrN phase results in the reduction in the corrosion resistance of the nitride layer in chromium-containing steels. The CrN phase is formed during nitriding above 500 °C and occurs frequently when using traditional methods of nitriding [1,2]. CrN precipitations, dissolved in ferrite, are very hard. The temperature increase causes them to undergo coalescence, which leads to the loss of coherence with the lattice, and the decrease in hardness, as well as initiate changes in the profile of long-range internal stresses. These changes already take place during nitriding. 

Depending on the nitrogen content, iron nitride phases with different structure and properties can be formed. Iron has five nitrides observed at ambient conditions, Fe_2_N, Fe_3_N_4_, Fe_4_N, Fe_7_N_3_ and Fe_16_N_2_ [15,19,20]. They are crystalline, metallic solids. Lately, it was put forward that the phase diagram of iron nitrides can be extended further, to even more N-rich compounds, such as the γ″-FeN and the γ‴-FeN phases [21].

All iron nitrides are metallic conductors and they are metastable with respect to decomposition into Fe and N_2_. The decomposition is limited by kinetic barriers. Atomic nitrogen can be dissolved in the body-centred cubic (bcc) lattice of α−Fe to a concentration of about 0.4 at% N without much distortion of the lattice. When more than 2.4 at% N is dissolved in pure Fe, the bcc lattice undergoes a tetragonal deformation. In the composition range up to about 11 at% N, the iron nitride compound is called nitrogen martensite α′. This phase has a body-centred tetragonal (bct) structure with lattice parameters depending on the nitrogen content. The N atoms occupy randomly octahedral hollow sites in the Fe sublattice. At saturation, nitrogen martensite has the Fe_8_N composition. The α′−Fe_8_N can transform into the α″-Fe_16_N_2_ phase. In this phase, the N atoms are ordered. It can be formed under special conditions from Fe, however, not in its pure form. The α″-Fe_16_N_2_ phase attracted considerable attention because of a possible very high saturation magnetization, reported to vary between 2.4 T and 3.2 T [16]. The next phase of the nitrogen content is the γ′-Fe_4_N phase (roaldite), which is cubic, with the Fe sublattice arranged in a face-centred cubic (fcc) structure and nitrogen atoms occupying the body-centred position 1/4. As indicated in the phase diagram, this phase has a narrow composition range around 20 at% N. The lattice parameter is 3.795 Å and the saturation magnetization was reported to be between 1.8 T and 1.9 T [17]. A saturation magnetization value in this range is slightly lower than the one of pure iron (2.21 T), making this phase somewhat less attractive in comparison with Fe. 

The authors of the paper [21] point to the important role of carbon in the process of phase changes in the nitride layer and its influence on the internal stress in this zone. Carbon diffusion implies a complex precipitation sequence and thermodynamical evolution that modify the volume change during nitriding. The transformation of initial carbides into nitrides decreases the kinetics of nitriding and is counteracted by the precipitation of cementite. Surface decarburization involves a decrease in the volume fraction of cementite during nitriding, leading to an unloading of the surface and thus reducing residual stresses. During nitriding, a surface was subject to mechanical loading–unloading through volume changes. The distribution of residual stresses is mainly governed by the thermochemical modifications due to nitrogen and carbon diffusion.

Studies show that the abrasion resistance of the nitride layer depends on the type of nitriding method [22]. Studies by G. Kugler et al. [23] have shown that the presence of a compound (white) layer protects the surface of dies against chemical reaction with hot aluminium [24]. It was found that annealing of the ε-Fe_3_N phase leads to the precipitation of the γ′-Fe_4_N phase. In this paper it was also found that long annealing of ε phase causes its homogenization. 

In general, it should be emphasized that any exposing a surface-nitrided tool to high temperatures leads to changes in the nitride layer. In combination with the changes that occur in the substrate, consisting in lowering the density of defects (recovery, polygonization) and the precipitation or decomposition of carbides, as well as their coagulation, this affects the properties of the nitride layer. In particular, the hardness and abrasion resistance of the nitrided surface change. Reducing these properties reduces the life of the dies and increases production costs.

The scope and scale of changes affecting the durability of the dies is of crucial importance due to the practice of using the dies in industry. From this point of view, the more stable the hardness of the nitrided layer is, the better the prognosis of the durability and utility of the die. 

Continuous development of the aluminium market causes the demand for dies to increase. According to the prediction of the International Energy Agency, between 2023 and 2030, the demand for aluminium will increase by more than 50 percent, due to, among other things, the rapid development of LED lighting and the development of the automotive industry [25]. The largest aluminium products’ production plant in Poland is Grupa Kęty S.A. S.A., which is a company with a long tradition [11]. Grupa Kęty S.A. assumes that, by 2019, it will become the leader in the production of aluminium profiles in Poland with sales of 1 364 million PLN in the Polish aluminium market [26]. 

The development of the aluminium industry generates the development of production and heat treatment of dies necessary for the production of aluminium profiles. Due to the importance of the die durability issue, the nitriding process is constantly being improved by companies producing nitriding furnaces. Therefore, all research on this subject contributes to the broadening of the knowledge on this subject and to the improvement of the nitriding technology.

In this paper the influence of temperature on the properties of the nitride layer of tool steel X37CrMoV51 (1.2344 tool steel) was analysed. Annealing was carried out for different time periods (8–60 h) and the influence of the given heat treatment conditions on the structural effects and properties of the nitride layer was evaluated.

## 2. Materials and Methods

The tested samples were nitrided in a Nitromax furnace under industrial conditions. 

Samples made from X37CrMoV51 steel were hardened and tempered, and then nitrided at 530 °C, according to Grupa Kęty S.A.’s (Kęty, Poland) proprietary technology based on the NITREG technology, under conditions in which industrial dies in Grupa Kęty S.A. S.A. are nitrided. During primary nitriding, a 120 to 140 µm thick diffusion layer should be formed on the dies. The thickness of the white layer should be between 4 and 6 µm and the hardness should be higher than 1000 units.

After nitriding, the samples were annealed in a furnace at 470 °C for 8 h, 12 h, 24 h, 30 h and 60 h, and additionally for 20 h at 270 °C. The samples were tested according to the following procedures for structure, hardness and abrasion resistance immediately after nitriding and again after annealing.

The research was carried out on cross sections. After cutting out the sample, it was sanded with grade #300 and #1200 abrasive papers and polished with diamond paste with grain sizes of 9 µm, 3 µm and 1 µm. The final polishing was carried out using the silica suspension OP-S according to Struers company procedures. In the next step, the samples were etched in Nital reagent with the following composition: 5 mL HNO_3_ + 100 mL C_2_H_5_OH.

The microstructure of the samples was examined using the Olympus GX-51 optical microscope (Cracow, Poland) and the Hitachi SU-70 scanning electron microscope (Cracow, Poland) equipped with Energy Dispersive X-Ray Analysis (EDX).

The microhardness of the nitride layer was examined using the Shimadz HMV-G apparatus (Cracow, Poland) by means of Vickers’ method with the load of 0.9807 N.

Chemical analyses in the microarea of nitrided steel and iron oxides formed as a result of gas nitriding were carried out using an electron probe microanalyser Jeol SuperProbe JXA-8230 (Cracow, Poland) equipped with Wavelength Dispersive X-ray analysis (WDS). The tests were performed in the Critical Elements Laboratory of AGH -KGHM at the Faculty of Geology, Geophysics and Environmental Protection of the AGH University of Science and Technology in Krakow. Investigations of nitrided steel were carried out with the use of an electron probe microanalyser with the acceleration voltage of 15 kV and the amperage of 50 nA, whereas, during the measurements of iron oxides, the amperage was 40 nA. The time of analysis of each element lasted 20 s in peak maximum position and 10 seconds in background position before and after the peak. The beam size was <1 µm. The following lines and standards were used for steel analysis: BN (NKα), Fe (FeKα), Mo (MoLα), V (VKα), Cr (CrKα). The following lines and standards were used for the analysis of iron oxides: BN (NKα), fayalite (FeKα), Mo (MoLα), V (VKα), Cr_2_O_3_ (CrKα). 

## 3. Results

**Figure 1:**
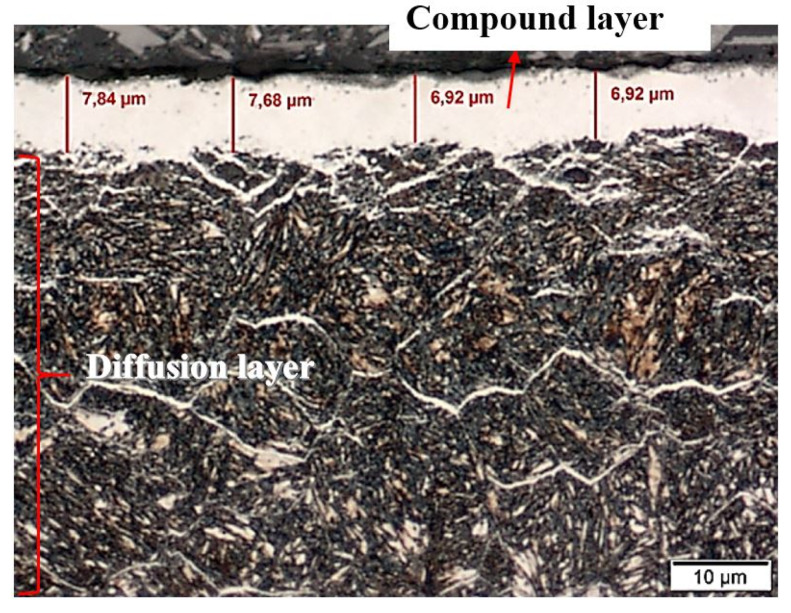
White layer in nitrided Nitromax furnace samples; microstructure of 1.2344 tool steel after gas nitriding shown though an optical microscope; white layer thickness ~7 µm, diffusion layer ~70 µm.

Studies on the structure of samples directly after nitriding showed that a white (compound) layer was formed on their surface (Figure 1). The thickness of this layer was estimated at about 7–8 μm, while the depth of the diffusion layer was about 70 μm (Figure 1).

**Figure 2:**
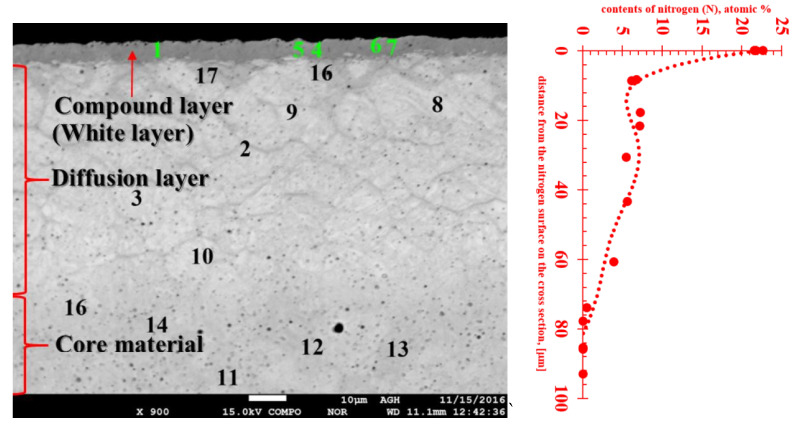
Point analysis of the chemical composition of: the white layer—points 1, 4–7; the diffusion layer—points: 2, 3, 8, 9, 10, 16, and 17; and the steel matrix—points 11–14 in 1.2344 steel after gas nitriding.

**Figure 3:**
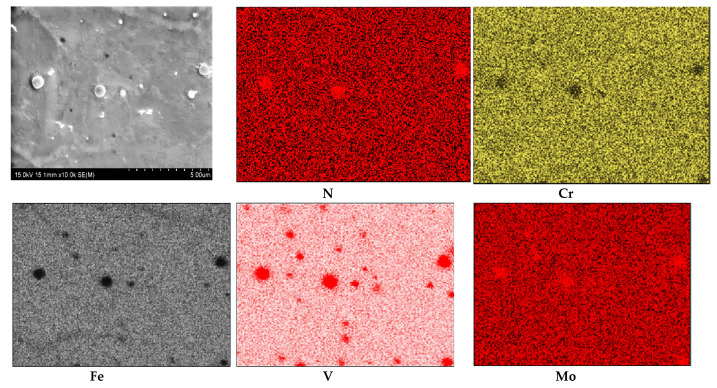
Map of elemental distribution in the white layer of the nitrided sample.

To estimate the nitrogen content in the studied layers the EDX method of chemical composition analysis with a scanning microscope was applied. The locations where the chemical composition was determined are shown in Figure 2 and quantitative results of chemical composition tests are shown in Table 1. The high nitrogen level and structural phase contrast identified a white (compound) layer. The white layer contained more than 21 at%. of nitrogen, which means the range of occurrence of γ′+ε, or ε-Fe_2_N nitride, γ′-Fe_4_N nitride and nitrogen ferrite, which contains 0.01% nitrogen at room temperature (Figure 3). Table 1 also shows results indicating the presence of a diffusion layer with nitrogen in the range 3.9–7.2 at%. These values indicate the extent of the presence of eutectoid mixture α+γ′ consisting of phases: γ‘-Fe_4_N nitride and α-nitride ferrite. 

The nitrogen profile attached to Figure 2 clearly shows that the very high nitrogen content in the near-surface layer decreases rapidly and already at a distance of about 10 µm from the sample surface, then at a distance of about 80 µm it reaches the zero level. This is the indicator of the boundary of the diffusion layer. Therefore, the thickness of the hard, nitride layer is relatively small, and the friction forces cause its rapid wear, which makes it necessary to regenerate the layer after a specified period of using the tool.

The results of WDS analysis of the chemical composition of the white layer (compound layer) in the form of elemental distribution maps are presented in Figure 3. In the maps, the position of nitrogen concentration appeared at the same place as the position of Fe, Cr, V and Mo. The average nitrogen content in the white layer was about 21.84 at% and in the diffusion layer it was about 5.82 at%. The average in both layers’ content of nitrogen was found to be 13.82 at%. The Cr content was estimated in white layer at about 4.71 at%, in the diffusion layer at about 5.35 at% and in the core material at about 5.75 at%—for an average value of about 5.27 at%. The content of Mo in the white layer was about 0.51 at%, in the diffusion layer about 0.66 at% and in the core material 0.71 at%. Generally the content of Mo was 0.63 at%. The V content in the white layer was about 0.75 at%, in the diffusion layer 2.03 at% and in the core material about 1.14 at%. The average V content was about 1.31 at%.

The existence of Cr, V and Mo in the same positions on the chemical composition maps from WDX measurement (Figure 3) suggests the probability of complex nitride precipitation. In such a case, chromium surely is dissolved in iron nitrides. It was shown that in ternary alloy systems where the crystal structures of the binary boundary nitrides are similar and the interaction parameter difference of the nitride forming elements is moderate, mixed ternary nitrides can develop [27]. Increasing numbers of ternary nitrides, A*_x_*M*_y_*N*_z_*, have been described in recent years, and these exhibit a great richness of structure and physical property [28,29].

**Figure 4:**
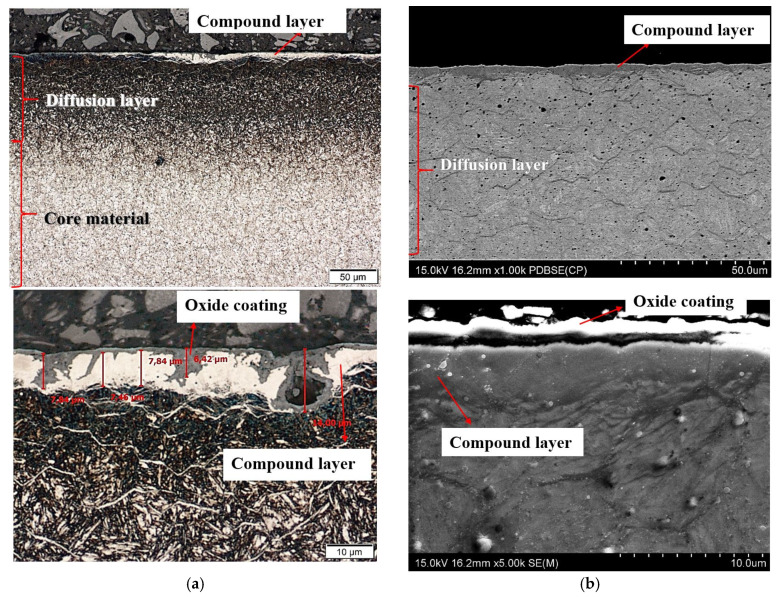
(**a**) Microstructure of WCLV steel after gas nitriding at Grupa Kęty S.A. and annealing for 3 h at 470 °C, show through an optical microscope (OM). Thickness of white layer ~7 µm and diffusion layer ~70 µm. Visible on the surface is the corrosive layer formed during annealing which results in degradation of the white layer (**b**) Microstructure of WCLV steel after gas nitriding in Grupa Kęty S.A. and annealing for 3 h at 470 °C, shown though a scanning microscope (SEM). Thickness of white layer ~7µm, diffusion layer ~70 µm and corrosion ~2 µm.

**Figure 5:**
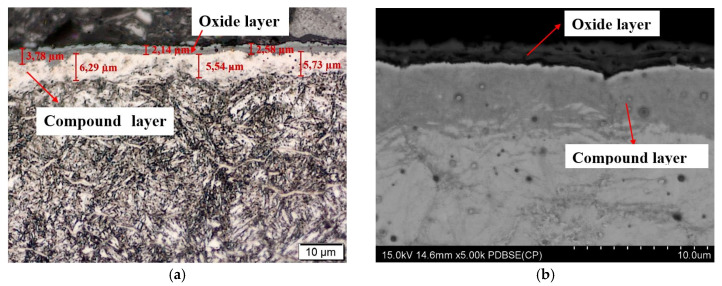
(**a**) Microstructure of WCLV steel after gas nitriding in Grupa Kęty S.A. and annealing for 8 h at 470 °C, shown with an optical microscope. Thickness of the white layer ~6 μm, diffusion layer ~70 µm and corrosion layer ~2 µm. The corrosion layer visible on the surface was formed during annealing, and it causes degradation of the white layer. (**b**) Microstructure of WCLV steel after gas nitriding in Grupa Kęty S.A and annealing for 8 h at 470 °C, shown with a scanning microscope. Thickness of white layer ~6 µm, diffusion layer ~70 µm and corrosion layer ~2 µm.

**Figure 6:**
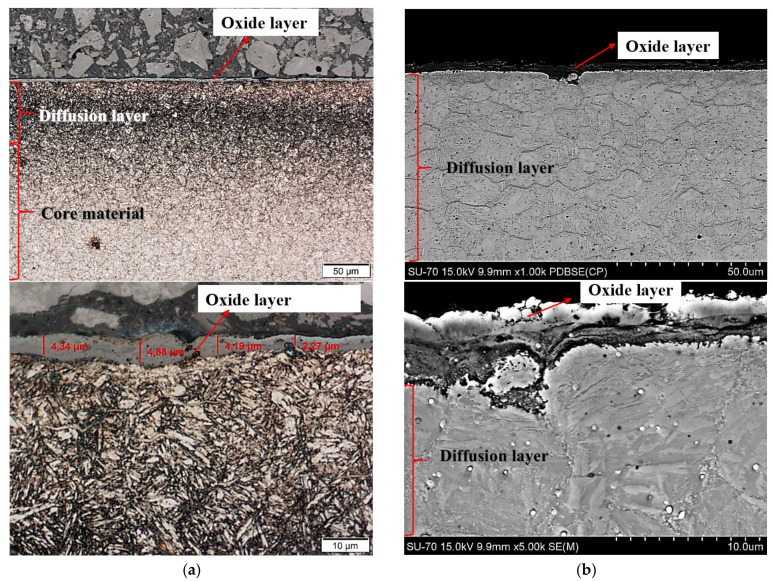
(**a**) Microstructure of WCLV steel after gas nitriding in Grupa Kęty S.A. and annealing for 30 h at 470 °C, shown with an optical microscope. No visible white layer. Layer thickness of diffusion layer ~80 µm and corrosion layer ~4 µm. (**b**) Microstructure of WCLV steel after gas nitriding in Grupa Kęty S.A and annealing for 30 h at 470 °C, shown with a scanning microscope. No white layer. Layer thickness of diffusion layer ~80 µm and corrosion layer ~4 µm.

**Figure 7:**
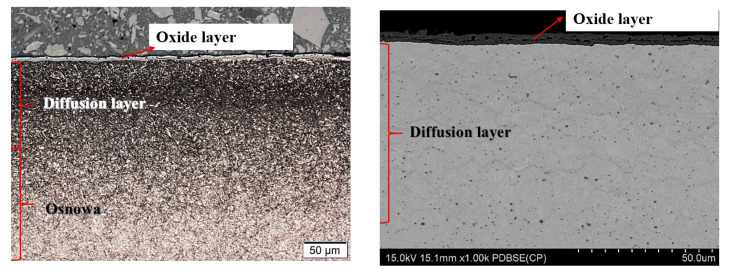

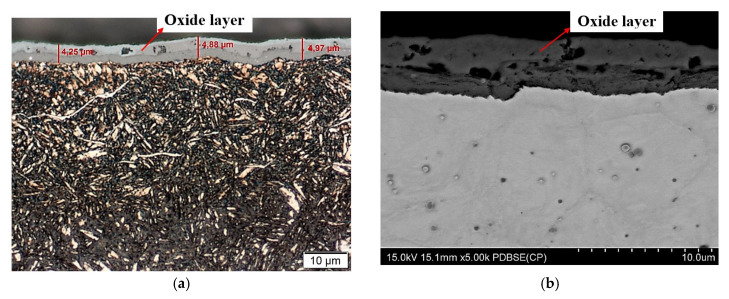
(**a**) Microstructure of WCLV steel after gas nitriding in Grupa Kęty S.A. and annealing for 60 h at 470 °C, shown with an optical microscope. No white layer. Layer thickness of diffusion layer ~100 µm and corrosion layer ~4.5 µm. (**b**) Microstructure of WCLV steel after gas nitriding in Grupa Kęty S.A. and annealing for 60 h at 470 °C, shown with a scanning microscope. No white layer. Layer thickness of diffusion layer ~100 µm and corrosion layer ~4.5 µm.

After nitriding, the samples were annealed at 470 °C for 3 h (Figure 4a,b), 8 h (Figure 5a,b), 30 h (Figure 6a,b) and 60 h (Figure 7a,b). As a result of annealing, the white layer was degraded. Microstructure observations show that the thickness of the white layer decreased as the annealing time increased, and for the annealing time of 30 h and 60 h it disappeared altogether. 

The width of the diffusion layer was observed to grow and equalled about 70 μm for 3 h and 8 h of annealing, about 80 μm for 30 h and about 100 μm for 60 h. 

The oxidation of nitride samples appeared locally after annealing for 3 h at 470 °C (Figure 4a) and increased with annealing time prolongation. The oxide growth at temperatures between 423 K and 623 K could be described consistently with the oxidation model of Fromhold and Cook (FC) [30]. It describes the initial oxidation of metals (0–20 nm). In the FC theory, the reaction between metal and oxygen takes place at the growing oxide/oxygen interface, requiring both metal ions and electrons to move through the oxide layer to the surface. The transport of electrons can proceed by two mechanisms: tunnelling and thermionic emission. For temperatures below 420 K, there is virtually no thermionic emission at the metal–oxide interface, and electron tunnelling is the dominant process. According to the FC formalism, at T ≤ 420 K, electron transport proceeds by tunnelling through the oxide layer and therefore the limiting film thickness should be nearly independent of the oxidation temperature. However, the measurements show that the saturation thickness does depend on the temperature and varies between 8.5 × 10^15^ O atoms/cm^2^ at room temperature (RT) and 15 × 10^15^ O atoms/cm^2^ at 395 K [31,32]. A two-layer system is formed, with a first layer containing Fe^2+^ only, and a top layer containing both Fe^3+^ and Fe^2+^. The growth of the second layer starts at an oxygen coverage of 4.0 × 10^15^ O atoms/cm^2^ and consists of Fe_0.77_O (which is probably a mixture of FeO and γ-Fe_2_O_3_). At higher oxidation temperatures, the relative fraction of Fe^3+^ in the formed oxide decreases. The oxide layer formed in O^2^ at 473 K consists of Fe^2+^ only. The decrease of the oxidation rate coincides with the formation of an oxide layer containing Fe^3+^. Upon annealing, Fe^3+^ is reduced to Fe^2+^, while the displaced Fe^0^ is oxidized to Fe^2+^. For larger oxide thicknesses (L > 3 nm) and higher temperatures (T > 420 K), the dominant electron transport processes thermionic emission. For higher temperatures and larger oxide thicknesses, the thermionic emission of electrons is rate-limiting. For lower temperatures and smaller oxide thicknesses the presence of Fe^3+^ drastically decreases the oxidation rate. The reduction rate of Fe^3+^ to Fe^2+^ increases with increasing temperature. An important feature of the FC theory is the concept of coupled currents: the net electrical current across the oxide layer is zero. At sufficiently high oxygen pressures, transport of either electrons or metal ions is rate-limiting.

The oxidation of nitride steel was investigated by Yang Li and all [33]. The iron oxide phases of hematite (Fe_2_O_3_) and magnetite (Fe_3_O_4_) through the post-oxidizing treatment at 400 °C and 450 °C was found. The hematite has been also identified in work [34] after the postoxidation of plasma nitriding AISI 4140 steel. In contrast, in [35], after oxidation of nitride layer at 480 °C to 500 °C, Fe_3_O_4_ (magnetite) was reported. It was also described that the precipitation of CrN or Cr in complex nitride precipitations at 500 °C removed Cr from the solid solution and adversely affected the oxidation performance at this temperature [35]. Formation of CrN and γ′-Fe_4_N, causes the corrosion resistance of the nitrided layer to decrease [36]. The nitrogen atoms occupy similar types of surface sites as the oxygen atoms [37] Displacement of N atoms from the surface into the bulk by interaction with gaseous O_2_ will be energetically rather favourable. The bulk diffusion coefficient of N in pure Fe has, at 900 K, a value of about 10^−7^ cm^2^/s and an activation energy of 18.9 kcal/mole. Extrapolation to room temperature yields a mean displacement by diffusion of about 5 A within 30 min. This process will, however, certainly be strongly accelerated by the rearrangement of the Fe atoms in the course of oxide formation (oxygen chemisorption on Fe(100)), which was found to cause an expansion of the topmost layers, as well as by the energy release associated with the oxygen attack so that displacement of the N atoms even at room temperature becomes feasible. On the other hand, the further growth of the oxide will certainly be retarded by previous formation of a nitride layer in the surface region—this is the well-known anticorrosive effect.

**Figure 8:**
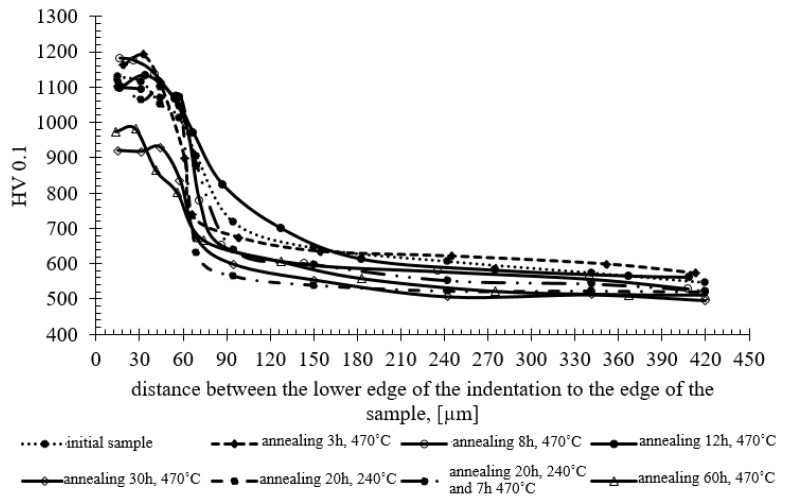
Hardness change profiles as a function of annealing time at 470 °C.

**Figure 9:**
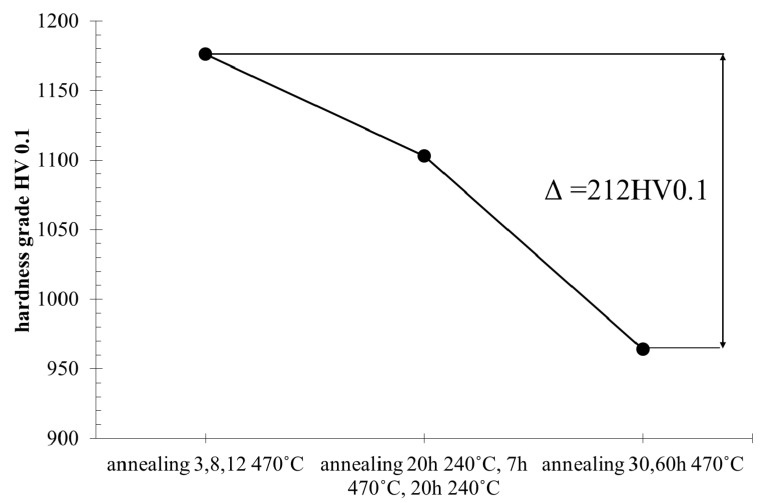
Mean hardness graph of three groups of annealed nitrided WCLV steel samples, measured at 15 µm from the sample surface. HV = 1176—mean hardness of sample group annealed at 3, 8 and 12 h at 470 °C; HV = 1103—mean hardness of sample group annealed for 20 h at 240 °C, 7 h at 470 °C and 20 h at 240 °C; and HV = 964—mean value of hardness for annealing at 30 h and 60 h at 470 °C.

The research showed that, as a result of long annealing times, the surface hardness of the samples decreased by about 200–250 units (Figure 8) in relation to the hardness of the original nitrided sample. The hardness results shown in Figure 8 can be divided into three groups, as presented at Figure 9. The highest hardness (average 1176 HV) is found in samples annealed for 3, 8 and 12 h at the temperature of 470 °C. The group of samples annealed for 20 h at 270 °C have the average hardness 1103 HV. The lowest hardness was found in samples annealed for 30 and 60 h at 470 °C with the average hardness 964 HV. The difference between the highest and lowest hardness is 212 HV.

**Figure 10:**
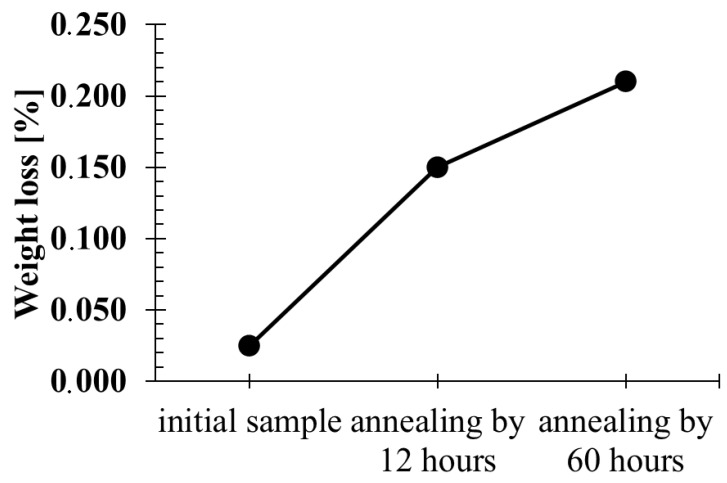
The weight loss of initial sample (0.025), sample annealed for 12 h in 470 °C (0.15) and samples annealed for 60 h at 470 °C (0.21).

Abrasion tests revealed a correlation between the hardness level and weight loss in the abrasion test. It was found that the lower the hardness, the more abrasive the surface of the samples was (Figure 10).

**Figure 11:**
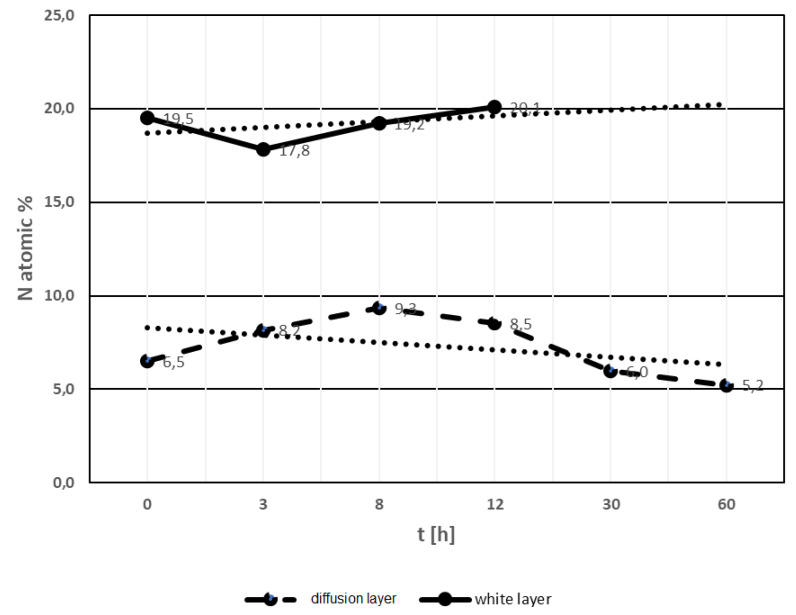
Mean nitrogen content in the white and diffusion layer in the samples after nitriding and annealing for 3–60 h at the temperature of 470 °C, measured at the same place from surface. The upper line shows mean value of nitrogen in white layer. The dotted line indicates estimation course in annealing time. The lower line indicates mean value of nitrogen content in diffusion layer and the dotted line indicates the tendency to decreasing nitrogen content with the extension of annealing time.

The analysis of nitrogen content in the white and diffusion layers was based on the results of EDX and WDS chemical composition tests. Figure 11 summarizes the data on nitride content in white and diffusion layers. The white layer contained on average about 19.4% atomic of nitrogen and this level of nitrogen is stable in samples annealed in the range of 3 to about 12 h at 470 °C. Above this time of annealing, the white layer disappeared. For the annealing times of 30 h and above, no white layer was found. The nitrogen content in the white layer suggests the occurrence of γ′ + ε phases. When the content of nitrogen decreases, the aforementioned phases do not exist. It should be assumed that the disappearance of the white layer occurred as a result of the decrease in nitrogen content, which diffused deep into the substrate, as evidenced by the increase in the thickness of the diffusion layer.

The diffusion layer contained about 8.1% atomic of nitrogen in samples annealed for 8–12 h (Figure 11). Samples annealed for more than 12 h showed an average nitrogen content reduced to about 5.6% atomic. Reduced nitrogen content in the diffusion layer predicted the presence of phases α + γ′.

## 4. Discussion

Coatings and layers produced by various surface engineering methods reveal a variety of structures and thicknesses. This is due to the mechanisms of forming coatings and layers. Nitriding is a specific surface treatment in which the top layer of a tempered tool is saturated with nitrogen at high temperatures. The effect of nitrogen in the atomic structure of steel is the formation of nitrides and the interstitial location of nitrogen in the crystal lattice. Depending on the concentration of nitrogen, different stoichiometric nitride compositions can be found. Apart from nitrides formed as a result of the compound of nitrogen and iron, i.e., nitrides such as Fe_2_N and Fe_4_N, moderate mixed ternary nitrides can also develop. In the conducted studies the searching of nitride particles were performed by EDX and WDS measurement of chemical composition in points and chemical composition maps. We found Cr, Mo, Fe, V and N concentration in the same places, which suggests formation in or quaternary phase [38,39]. The investigation in work shows that at lower Cr/Mo ratio, hexagonal CrMoN_2_ precipitation occurred.

The presence in the nitride test layer of a hard nitrogen phases occur near the surface of the samples is significant for die resistance to abrasion. Nitrogen phases especially increase the hardness, which translates into higher abrasion resistance [40]. Reducing the surface nitrogen content in the white layer leads to phase changes and loss of high hardness [41,42]. As a result of experimental annealing, the initial hardness of the nitride layer, amounting to approximately 1190HV, was reduced by approximately 210 units (Figure 8). From a practical point of view, reducing the hardness below 1000HV disqualifies aluminium extrusion dies for further use. During the annealing at 470 °C, the oxidation of the sample surface develops. The thickness of the oxide layer increases with annealing time prolongation. However, above 8 h of annealing, it becomes almost stable. Experimental investigations show a “parabolic law” of oxide layer growth [43]. Dwivedi D., Lepková K. and Becker T. [44] point out on the importance of texture in the corrosion process. Robert E. Melchers [45] presented different models of corrosion loss starting from different assumptions and showed parabolic and linear lows of oxidation. The oxidation zone containing (Mn,Fe)O and (Mn,Cr,Fe)_3_O_4_ oxides in annealed Fe–1.9Mn–1.6Cr steel was investigated by Mao, W., Ma, Y. and Sloof, W.G [46]. It was found that the penetration depth of oxides along grain boundaries is much larger (by a factor of two or more) than that of internal oxides formed inside grains. They also found a parabolic rate law of oxidation. In relation to obtained results, it should be assumed that the parabolic oxide layer grows and oxidation stabilizes in the range of 30 h to 60 h of annealing.

It should be mentioned that the presence of nitride and carbide phases in tools for which no additional heat treatment is applied is beneficial due to the high surface hardness. However, if the surface of the die is designed to be covered with an additional coating, e.g., PVD - Physical Vapour Deposition, the heat treatment should be carried out in such a way that no white layer is formed. There should only be a diffusion zone characterized by maximum effective thicknesses and devoid of carbide precipitations at the grain boundaries of the former austenite [42]. It can therefore be concluded that the design and implementation of the nitriding process and its effects will depend on the purpose for which the tools are used.

The life cycle of the dies includes periods at elevated temperatures (470 °C and 520 °C) and periods at room temperature. Cyclic changes in the working temperature during die life of the die make it subject to processes of structural and phase changes. This results in changes of properties and ultimately, after the loss of the required hardness level, the need for regeneration through secondary nitriding.

The substrate is also subject to changes under the influence of temperature. There are recovery processes that reduce dislocation densities and other lattice defects. The processes of precipitation coagulation take place. Precipitations that were coherently bound to the matrix lose their coherence, which results in a decrease of hardness. The phenomena of structure restoration, combined with changes in the morphology of the precipitations, lead to unfavourable changes of properties in terms of die exploitation. 

## 5. Conclusions

On the basis of the research carried out, it was found that:

1. Annealing of the nitrided samples leads to degradation of the nitride layer and the decrease of hardness.
2. It was found that annealing of the samples at 470 °C, over 12 h causes a decrease in mean hardness value from 1176 HV to 1103 HV and annealing the samples over 30 h at this temperature leads to a decrease in hardness to 964 HV.
3. Tests have shown a decrease in abrasive resistance as the annealing time increases.
4. Annealing over 30 h at 470 °C caused the white layer to disappear and the average nitrogen content in the diffusion layer to decrease to the level of about 6 at%.

## Figures and Tables

**Table 1:** Results of chemical composition analysis in the points marked in Figure 2.

	N	Mo	Fe	V	Cr	Kind of Nitride Layer
	Atomic %					
2	5.442	0.697	84.946	0.961	5.237	Diffusion layer
3	5.532	0.703	85.036	0.980	5.432	
4	21.702	0.499	70.824	0.582	4.606	Compound layer (white layer)
5	21.563	0.557	70.355	1.220	4.572	
6	22.615	0.488	69.383	0.627	5.062	
7	21.513	0.514	71.019	0.577	4.587	
8	7.201	0.601	83.927	0.727	5.295	Diffusion layer
9	7.117	0.597	84.045	0.698	5.256	
10	3.823	0.693	86.829	0.780	5.528	
11	0.000	0.695	90.185	0.887	5.829	Core material
12	0.000	0.710	90.165	0.961	5.750	
13	0.000	0.721	90.287	0.901	5.635	
14	0.000	0.717	90.171	0.906	5.797	
15	0.452	0.737	89.747	1.009	5.709	Boundary between diffusion layer and core material
16	6.698	0.583	84.632	0.761	5.264	Diffusion layer
17	6.117	0.635	84.690	0.715	5.684	
